# Characterization of a novel murine Sost ER^T2^ Cre model targeting osteocytes

**DOI:** 10.1038/s41413-018-0037-4

**Published:** 2019-02-21

**Authors:** Delphine B. Maurel, Tsutomu Matsumoto, Julian A. Vallejo, Mark L. Johnson, Sarah L. Dallas, Yukiko Kitase, Marco Brotto, Michael J. Wacker, Marie A. Harris, Stephen E. Harris, Lynda F. Bonewald

**Affiliations:** 1 Department of Oral and Craniofacial Sciences, University of Missouri-Kansas City School of Dentistry, Kansas City, MO USA; 2 Department of Anatomy and Cell Biology, Indiana University School of Medicine, Indianapolis, IN USA; 3 Bone−Muscle Collaborative Sciences, College of Nursing and Health Innovation, University of Texas at Arlington, Arlington, USA; 4 Department of Biomedical Sciences, University of Missouri-Kansas City School of Medicine, Kansas City, MO USA; 5 University of Texas Health Science Center, San Antonio, TX USA; 6 Department of Orthopaedic Surgery, Indiana University School of Medicine, Indianapolis, IN USA; 7 Present Address: Pharmaceutical Sciences Department, Universite de Bordeaux, Bio-Tis, INSERM Unité 1026 BioTis, 146 Rue Léo Saignat, 33076 Bordeaux, France

**Keywords:** Bone, Metabolism

## Abstract

Transgenic mice are widely used to delete or overexpress genes in a cell specific manner to advance knowledge of bone biology, function and disease. While numerous Cre models exist to target gene recombination in osteoblasts and osteoclasts, few target osteocytes specifically, particularly mature osteocytes. Our goal was to create a spatial and temporal conditional Cre model using tamoxifen to induce Cre activity in mature osteocytes using a Bac construct containing the 5’ and 3’ regions of the *Sost* gene (Sost ER^T2^ Cre). Four founder lines were crossed with the Ai9 Cre reporter mice. One founder line showed high and specific activity in mature osteocytes. Bones and organs were imaged and fluorescent signal quantitated. While no activity was observed in 2 day old pups, by 2 months of age some osteocytes were positive as osteocyte Cre activity became spontaneous or ‘leaky’ with age. The percentage of positive osteocytes increased following tamoxifen injection, especially in males, with 43% to 95% positive cells compared to 19% to 32% in females. No signal was observed in any bone surface cell, bone marrow, nor in muscle with or without tamoxifen injection. No spontaneous signal was observed in any other organ. However, with tamoxifen injection, a few positive cells were observed in kidney, eye, lung, heart and brain. All other organs, 28 in total, were negative with tamoxifen injection. However, with age, a muscle phenotype was apparent in the Sost-ER^T2^ Cre mice. Therefore, although this mouse model may be useful for targeting gene deletion or expression to mature osteocytes, the muscle phenotype may restrict the use of this model to specific applications and should be considered when interpreting data.

## Animal model: Transgenic mice offer platform to study mature osteocytes

A new transgenic mouse allows DNA modifications to be targeted to mature bone cells known as osteocytes, enabling researchers to better study the function of this cell population, although the model has some weaknesses that may restrict its use. Lynda Bonewald from Indiana University, Indianapolis, USA, and colleagues engineered mice to express introduced genetic material only in cells in which a gene called *Sost* is normally active — namely, osteocytes — and only when experimenters introduce a drug called tamoxifen. In general, transgene activity was contained to the osteocytes, but a full characterization of the mice showed some issues. For one, spontaneously transgene expression occurred in older mice in the absence of tamoxifen, although this was limited to the mature bone cells. With tamoxifen, the researchers observed some transgene expression outside of the bone. Most problematically, there were some broad muscle defects.

## Introduction

The osteocyte was thought to be a passive cell, but in the last decade it has been shown to have many functions, such as regulation of bone formation and resorption, generation of endocrine factors that target kidney and muscle and mechanotransduction (reviewed in^1^). Today it is accepted that the osteocyte, the most abundant cell in bone, is a key player in bone regulation and adaptation to loading or unloading.^2,3^ Based on recent research, new drugs are being developed such as anti-sclerostin antibody that appears promising in animal models^4^ and is now being tested in humans.^5^ Other factors made by osteocytes, which are the focus for therapeutics, are RANKL^6^ and FGF23.^7^

In contrast to osteoblasts and osteoclasts, there are very few Cre animal models targeting osteocytes specifically.^8–11^ The first osteocyte-selective cis regulatory region used to target osteocytes and late osteoblasts was Dentin Matrix Protein-1 (Dmp1) based on the studies of Yang and colleagues on the 10, 8, and 2.5 kb promoter regions.^12^ The Dmp1-Cre mouse model was developed by these same investigators using the 10 kb fragment of the Dmp1 promoter (−9 624- + 4 439).^13^ Cre activity was determined using a Rosa26R reporter mouse in which LacZ expression is activated following excision of a stop cassette. Reporter expression was mainly observed in osteocytes with low expression in osteoblasts. However, when this mouse was crossed with the Ai9 mouse in which the recombination event causes activation of tdTomato fluorescent protein (Jackson Labs), fluorescence was detected in several other tissues including muscle, brain, kidney, and the majority of osteoblasts on the bone surface.^9,10^ Gorski and colleagues found that the Cre recombination in muscle was dependent on the animal.^14^ Unintended targeting of Dmp1-Cre showed an effect on the gastrointestinal mesenchyme.^15^ It is not clear why this variability in Cre activation occurs but it may be due to mouse strain and/or spontaneous activation during various stages of development.

Other attempts have been made to produce a more osteocyte specific Cre mouse model using the 8 kb Dmp1 promoter.^16^ However, in this mouse, Cre activity was observed on endocortical and cancellous surfaces of bone.^9^ To avoid non-specific activity of Dmp1-Cre in developmental stages, a tamoxifen-inducible Cre was generated in which a mutated estrogen ligand-binding domain was used.^17^ In this mouse model, some spontaneous (non-tamoxifen induced) Cre activity was observed in osteocytes, but not in osteoblasts. Low tamoxifen targeted mainly osteocytes while high tamoxifen levels also activated Cre in osteoblasts.^9^ No activity was observed in muscle, perhaps because of no expression during developmental stages.

Other osteocyte selective markers in addition to Dmp1 have been identified, including Phex, E11/gp38, Mepe, FGF23, and Sost.^1^ Dmp1, Phex, and E11 are mainly expressed in early osteocytes while Mepe, FGF23, and Sost are expressed in mature osteocytes. Sclerostin is a secreted cysteine^−^knot protein among the DAN family, and is encoded by the Sost gene.^18^ It was discovered in 2001 while studying a mutation that caused the disease sclerosteosis, where family members had massive bone overgrowth. Sost mRNA is expressed in many tissues during embryogenesis in humans, including heart, aorta, liver and kidney.^18–20^ In the adult, it has been reported only in terminally differentiated cells encased in a mineralized matrix, osteocytes, hypertrophic chondrocytes and cementocytes.^21–24^

Recently a Sost-Cre has been generated which leads to recombination in osteocytes but not osteoblasts or lining cells.^25^ However, large numbers of fluorescent cells are present in bone marrow and some osteoclasts are positive, suggesting that Cre recombination occurred during development in hematopoeitic precursor cells. As there is a critical need for additional osteocyte selective Cre models, we focused on creating a new Cre ER^T2^ model, driven by the Sost promoter. Our hypothesis was that Sost ER^T2^ Cre would be expressed only in mature osteocytes in bone and not in other tissues and therefore would be useful for future research targeting osteocyte expression and functions. Here we describe this new Cre mouse model for targeting osteocytes.

## Results

### Generation of Sost ER^T2^ mice

**Fig. 1:**
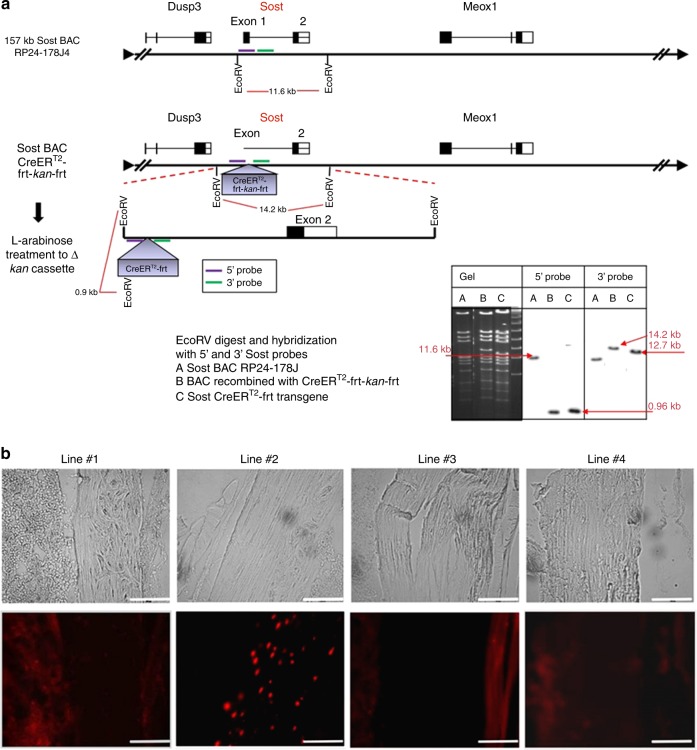
Generation of Sost ER^T2^ Cre mice. **a** A Sost Bac construct containing the 5’ and 3’ regions of the Sost gene (Sost ER^T2^ cre) was made, using RP24-178J4 BAC (157Kb) with the Sost gene. Recombineering was used to replace the ATG region with the CreER^T2^ frt-kan-frt cassette, and the frt-kan-frt cassette was then removed with I-arabinose inducible flip recombinase in the SW105 bacteria. The digest with EcoRV shows a 11.6 kb band that is only 0.9 kb after insertion of the CreERt2-frt-kan-frt cassette, using the 5’ probe(purple). After removing the kan cassete the EcoRV band goes from 14.2 kb to 12.7 kb with the 3’ probe (bottom right of 1 a). **b** Representative images from the four founder lines (#1, #2, #3, #4) that were crossed with Ai9 tdTomato mice to analyze the DNA recombination in osteocytes after tamoxifen injection (300–555 mg·kg^−1^). Images of the femur were taken at 20x magnification. The upper panels are brightfield images and the lower panels show TdTomato fluorescence. Scale bar represents 100 µm. Line #2 appeared the most promising and osteocyte specific as compared to the other lines

Sost-ER^T2^ cre mice were generated in order to create a spatial and temporal conditional Cre model using tamoxifen to induce Cre activity in osteocytes. This has been done by using the Sost Bac construct containing the 5’ and 3’ regions of the Sost gene (Sost ER^T2^ cre). Using RP24-178J4 BAC (157Kb) with the Sost gene, recombineering was used to replace the ATG region with the CreER^T2^ cassette, and the frt-neo-frt cassette was then removed with L-arabinose inducible flip recombinase. (Fig. 1a). Founder lines on a C57Bl6 background were created by pronuclear injection of the SostBAC-CreER^T2^ construct. These lines are viable and fertile. Four founder lines (Line #1, #2, #3 and #4) were then crossed with the Rosa26-loxP-stop-loxP-tdTomato or Ai9 allele (B6;129S6-*Gt(ROSA)26Sor*^*tm9(CAG-tdTomato)Hze*^/J reporter mouse to analyze activity of the Cre transgene in bone in the presence and absence of tamoxifen and to determine any off target activity of the Cre transgene.

### Cre activity in bone following tamoxifen injection

Out of the four transgenic lines, only line #2 showed strong and consistent Cre recombination in osteocytes following tamoxifen treatment (Fig. 1b). Line #1 and line #4 showed few positive osteocytes as did line #3, which also showed off target activity in other tissues/cells. Therefore, strain #2 was selected for further characterization.

### Cre activity in bone using Line #2

**Fig. 2:**
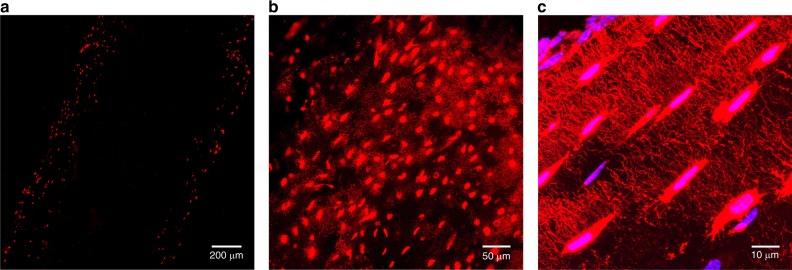
Specificity of Cre activity in osteocytes and dendritic processes in Sost ER^T2^ Cre mice. In bone, the tdTomato signal showed strong specificity for osteocytes in Sost ER^T2^ Cre/tdTomato mice treated with tamoxifen, as no signal was seen in osteoblasts, muscle or bone marrow. **a** Epifluorescence microscopy in the femur at 4x magnification. Higher power **b** (20 × ) and **c** (100 × ) confocal images are shown of the lacunocanalicular network in the femur of these mice showing specific targeting of Cre activity to osteocytes and also showing that these mice may be useful to analyze the osteocyte network as well as their dendrites and connections. TdTomato is shown in red and DAPI signal is in blue

In bone, as determined using the Sost Cre-ER^T2^ tdTomato reporter mouse, Cre was expressed specifically in osteocytes after tamoxifen injection. In the Cre^+^ mice, fluorescence was only observed in osteocytes (Fig. 1b Line #2 and Fig. 2a) with no fluorescence in osteoblasts, osteoclasts, bone marrow or muscle. Expression in femurs with tamoxifen injection in 2 mo old females was (15.3 ± 6.3)% (*n* = 4), in 2 mo old males was (52 ± 2)% (*n* = 2), in 5 mo old females it was (28.0 ± 7.4)% (*n* = 4) and (40.5 ± 17.8)% in 5 mo old males (*n* = 4). Occasionally a very few chondrocytes were positive in adult mice (data not shown). It has been described previously that hypertrophic chondrocytes can express sclerostin.^22^ The osteocyte lacuno-canalicular network could also be visualized in these mice using confocal microscopy (Fig. 2b, c).

### Spontaneous Cre activity in the absence of tamoxifen injection

**Fig. 3:**
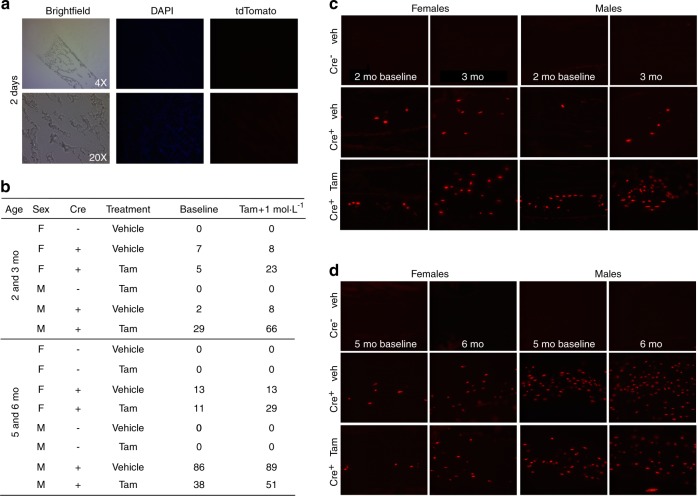
Examples of spontaneous Cre activity in some osteocytes with age and induction of more osteocyte-specific Cre activity with tamoxifen. **a** Brightfield, DAPI and TdTomato images of the femur in 2 day old Sost ER^T2^ Cre/tdTomato mice without tamoxifen treatment at 4x and 20x magnifications show no spontaneous Cre activity. **b** Percentage of positive osteocytes in tail vertebrae in single animals at 2 mo before injection of tamoxifen (Baseline) and at 3 months of age after tamoxifen injection (Tam + 1 mol·L^−1^) and at 5 mo before injection of tamoxifen and 6 mo of age after tamoxifen injection is shown. The 3–5th caudal tail vertebra in females and males in Cre^–^ control mice, Cre^+^ injected with vehicle, Cre^+^ injected with tamoxifen are shown. Some spontaneous induction of Cre activity occurs in the absence of tamoxifen. Cre-mediated recombination was higher in males than in females. One 5 mo old male animal showed 86% baseline or spontaneous Cre activity. **c** and **d** Images of the tdTomato fluorescent reporter to indicate Cre-mediated recombination in the tail vertebrae of male and female Sost ER^T2^ Cre/tdTomato mice with and without tamoxifen treatment at baseline and 1 month after tamoxifen injection (**c**. 2 mo and **d**. 5 mo). Cre^–^ controls with vehicle injection are also shown. These images are from the same animals as shown in 3**b**. The scalebars represent 100X

Surprisingly, a background level of spontaneous Cre activation was observed without tamoxifen injection in Sost Cre-ER^T2^ tdTomato mice at 2 and 5 mo of age. Because of this finding, 2 day old and one mo old mice were also examined to determine when this spontaneous (“leaky”) activity started. No fluorescence was observed in bones or in any soft tissues without tamoxifen at 2 days of age (Fig. 3a). Tail vertebrae were then examined in 1 and 2 mo old Sost Cre-ER^T2^ tdTomato mice at baseline and after tamoxifen injection. In females, the percentage of positive osteocytes was approximately 6% at 1 mo and increased to around 12% at 2 mo of age. In males, the ‘leakiness’ was about 2% at 1 mo compared to about 31% at 2 mo of age. No fluorescence was ever observed in Cre^–^ mice.

### Tamoxifen injection increased Cre activity

The spontaneous Cre activity did not appear to increase significantly after 2 months of age in tail vertebra (Table 1). One month after tamoxifen injection of 2 mo old or 5 mo old Cre^+^ mice, the percentage of Cre expressing osteocytes was increased in females and in males compared to vehicle control injected animals (Fig. 3 and Table 1). Tamoxifen appeared to double the number of positive osteocytes (Table 1). It was interesting that Cre recombination was observed more extensively in males than in females, which is consistent with the expression of Sost in bone.^26,27^ There was also variability in the amount of Cre activity between littermates as one unusual animal had an 86% expression at 5 months before the injection of tamoxifen (Fig. 3b, c). Both the baseline (without tamoxifen) and inducible (with tamoxifen) Cre activity in bone was highly specific to osteocytes, with no Cre activity in osteoblasts, bone marrow or muscle.

**Table 1:** Percent positive osteocytes in tail vertabrae in male and female Cre^+^ mice with and without tamoxifen (250–550 mg·kg^−1^) injection. The basal Cre activity or ‘leakiness’ does not appear to increase after 2 mo. (Mean plus/minus standard error)

Sex	Age	Basal Activity or “leakiness”	With Tamoxifen	Sample size
Female	2 mo	11.5 ± 6.4	25.0 ± 4.1	4
	5 mo	13.0 ± 2.2	24.3 + 4.1	3
Male	2 mo	31 ± 8.3	74.3 ± 14.7	3
	5 mo	31 ± 13.1	53.4 ± 7.6	4

### A small amount of off target Cre activity was observed in non-bone tissues

**Fig. 4:**
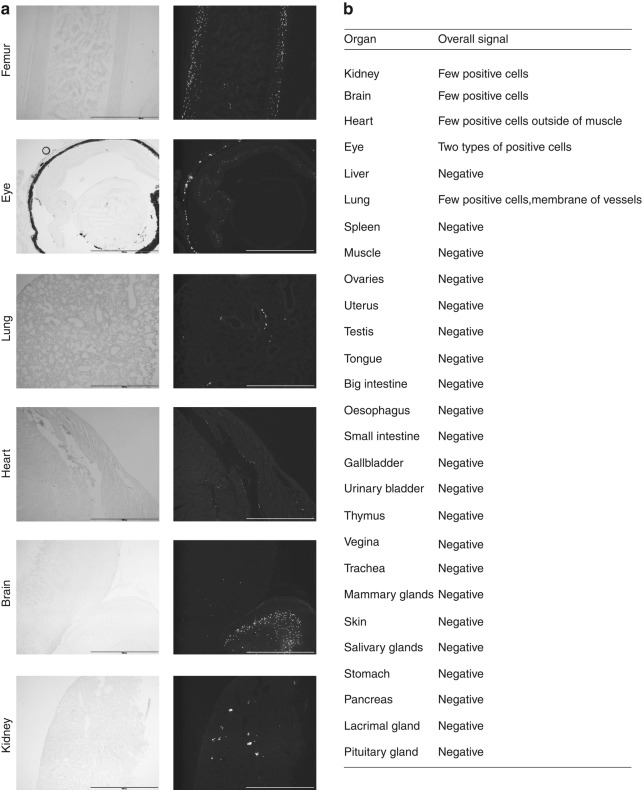
Off target Cre activity only with tamoxifen injection of Sost ER^T2^ Cre/tdTomato mice. **a** Positive Cre activity in femoral bone for comparison to off target expression in cells in the eye, lung, heart, brain and kidney. Males 2 mo old are shown for femur and heart and males 5 mo old are shown for eye, lung, brain, and kidney. Left panels show brightfield images and right panels show TdTomato fluorescence. Scale bar represents 1 000 µm. **b** All the other organs and glands examined (22 in total) for off target Cre activity were negative

To determine the specificity of Cre activity and identify any off target activity in the Sost Cre-ER^T2^ tdTomato model, the tdTomato fluorescence was examined in an extensive set of 27 different soft tissues. No signal was observed in any of the soft tissues in any Cre^–^ mice nor in the Cre^+^ mice without tamoxifen (data not shown). Off target effects were only observed in Cre^+^ mice injected with tamoxifen. With tamoxifen injection, fluorescence was detected in a very small percentage of cells in the eye, kidney, heart, lung and brain (Fig. 4a). All the other tissues analyzed were negative for Cre activity (Table in Fig. 4b). In lung and heart, the signal appeared to localize with blood vessels. Some cells in the glomerulus were positive in kidney, and the retina and choroid in the eye. A very small and specific region of the brain was positive. We also performed immunostaining for sclerostin in these tissues but signal was only detectable in osteocytes (data not shown) and not in the other tissues even those with Cre activity as indicated by the tdTomato reporter.

### Bone and muscle phenotype in Sost-CreER^T2^ mice in the absence of floxed gene recombination

**Fig. 5:**
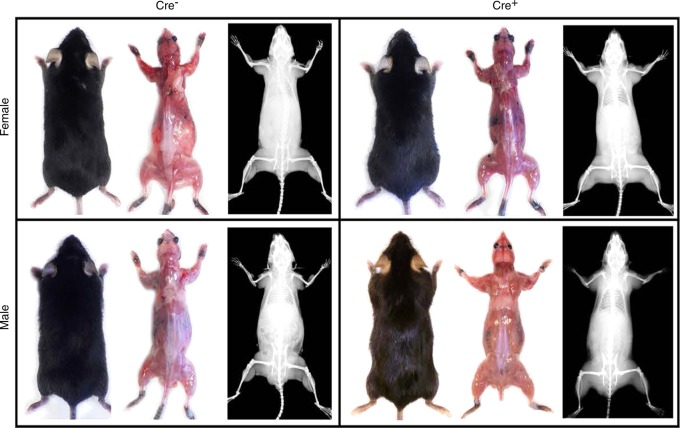
Images of 6 mo male/female intact, muscle, and X-ray plus table of micro CT results. Images of 6-month-old female and male mice, Cre^–^ control and Sost-CreER^T2^ /tdTomato mice w/wo skin. The x-ray images were of the same mouse. None of the mice had been injected with tamoxifen.

We next examined the Sost-CreER^T2^ mice without crossing them with reporter lines or floxed lines to determine whether there were any effects of the Sost-CreER^T2^ transgene. At 6 months of age no visible, external differences could be observed between wildtype Cre^−^ and Sost-CreER^T2^ male and female mice, nor significant visible differences in muscle mass or bone mass by x-ray (Fig. 5). There were also no significant differences in body weight between Cre^−^ and Sost-CreER^T2^ in males or females at 1, 3, or 6 mo of age nor were there any significant differences in bone parameters except in trabecular thickness in males where the Sost-CreER^T2^ were significantly wider than the Cre^−^ (Table 2).

**Table 2:** Micro CT analysis of trabecular bone and cortical bone in tibia and whole body weight. (*n* = 4–5)

Bone parameters		Cre^-^		Cre^+^		*P* value	
		Female	Male	Female	Male	Female	Male
Trabecular bone analysis	(Tb.BV/TV)/%	0.083 ± 0.013	0.088 ± 0.023	0.112 ± 0.031	0.127 ± 0.034	0.093	0.116
	Tb.N/µm^-1^	1.773 ± 0.252	4.175 ± 0.473	2.406 ± 0.568	3.953 ± 0.366	0.052	0.487
	Tb.Th/µm	0.045 ± 0.002	0.044 ± 0.002	0.045 ± 0.004	0.054 ± 0.006	0.843	*0.024
	Tb.Sp/µm	0.438 ± 0.235	0.238 ± 0.030	0.389 ± 0.102	0.247 ± 0.026	0.678	0.676
Cortical bone analysis	(Ct.BV/TV)/%	0.916 ± 0.003	0.915 ± 0.006	0.915 ± 0.004	0.915 ± 0.002	0.790	0.977
	Ct.Th/µm	0.212 ± 0.002	0.211 ± 0.014	0.208 ± 0.003	0.210 ± 0.009	0.100	0.870
Body Weight	Gram	31.70 ± 2.574	28.00 ± 3.317	31.75 ± 2.111	26.26 ± 2.353	0.977	0.367

No significant differences were observed between control Cre^–^, mice, compared to Sost-CreER^T2^ mice/tdTomato, of the same gender using unpaired Student’s *t*-test *P < 0.05

**Fig. 6:**
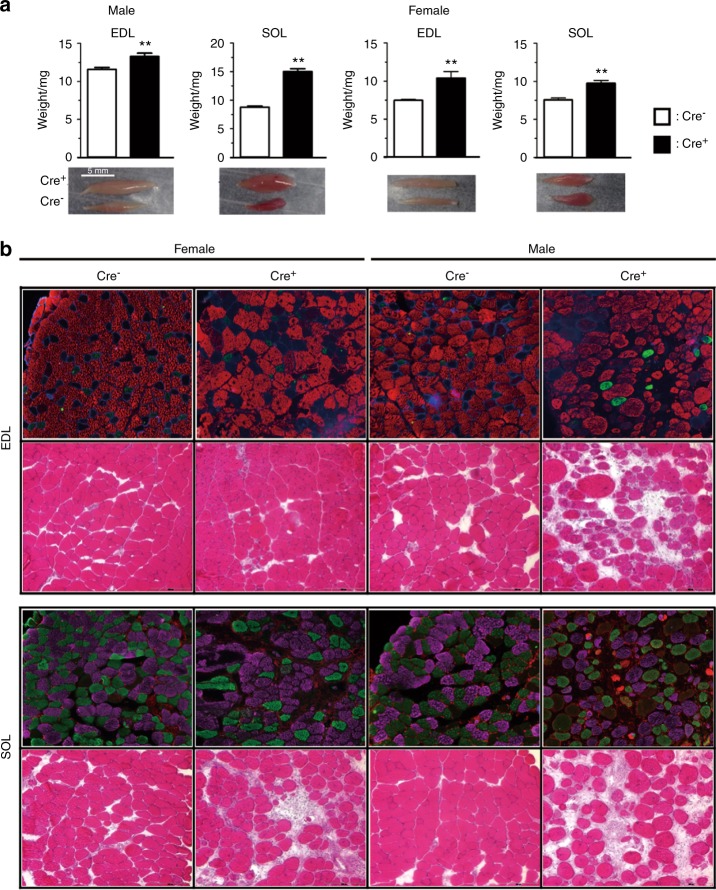
Excised muscle and histology from 6 mo male and female Cre^−^ and Cre^+^ animals. **a** Image and weight of EDL and SOL muscle from control Cre^−^ mice and Sost-CreER^T2^, Cre^+^, mice at 6 months of age. **b** Muscle fiber typing by immunohistochemistry and Hematoxylin-Eosin stain for EDL and SOL muscle sections from control Cre^–^ mice and, Sost-CreER^T2^ Cre^+^ female and male mice. Myosin type I fibers; violet, type IIa fibers; green, type IIb fibers; red were representative fluorescent colors. Significantly different from control wild type mice of the same gender using unpaired Student’s *t-*test ** *P* < 0.01

In 1 and 3 month old males, SOL, EDL, and gastrocnemius muscles from Sost-Cre^−^ ER^T2^ were heavier than wildtype controls (in mg): 1 mo SOL 6.1 ± 1.4 vs. 4.5 ± 0.3; 1 mo EDL 6.1 ± 0.8 vs. 5.2 ± 0.3; 1 mo gast. 99.1 ± 20.6 vs. 75.4 ± 2.1; 3 mo SOL 12.3 ± 1.1 vs. 8.9 ± 1.2; 3 mo EDL 12.2 ± 1.2 vs. 10.6 ± 1.0; 3 mo gast. 200.2 ± 22.4 vs. 155.2 ± 11.0 (*P* < 0.05; *n* = 6 for all muscles). Interestingly, in 1 month old females, SOL and gastrocnemius muscle weighed less in Sost-CreER^T2^ compared to wildtype controls (in mg): SOL 4.5 ± 0.2 vs. 5.5 ± 0.5; gast. 68.0 ± 9.7 vs. 83.1 ± 4.2 (*P* < 0.05; *n* = 4–6) with no difference in EDL, 5.3 ± 0.1 vs. 5.5 ± 0.3 (*P* > 0.05; *n* = 4). At 3 months of age, there was no significant difference in female muscle weights: SOL 7.9 ± 1.9 vs. 6.9 ± 0.4; EDL 9.9 ± 2.7 vs. 7.9 ± 0.4; gast. 138.4 ± 26.3 vs. 119.7 ± 3.1 (*P* > 0.05; *n* = 6). By 6 mo of age, the EDL and SOL from both female and male Sost-CreER^T2^ mice were significantly heavier than wildtype Cre^−^ controls (Fig. 6a).

Histological characterization of myosin fiber type in 6 month old EDL and SOL of Sost Cre^−^ER^T2^ mice was performed by IHC and H-E staining. The characteristic arrangement of muscle fibers (i.e., polygonal shaped fibers, relatively small variation in size, peripheral nuclei, and scant endomyosial collagen) was disrupted in Sost-CreER^T2^ compared with control. The Sost-CreER^T2^ muscles appeared myopathic, particularly in males. By H&E staining, most of the muscle cellular nuclei in Sost-CreER^T2^ were centralized, and muscle fibers appeared more scattered, rounded, and grouped with significantly more collagen indicating fiber replacement by fibrosis; all key characteristics of myopathy. Normally with myopathy, fibers show atrophy; however, in this case, perhaps in adaptation to the overall muscle hypertrophy, the muscle fibers are not atrophic, particularly in the SOL muscle. (Fig. 6b). Also interesting was that despite the myopathic features, the Sost-CreER^T2^ muscle fibers did not show any splitting.

Moreover, our fiber typing by immunostaining suggests that in both male and female Sost-CreER^T2^ EDL muscles, larger areas of purple indicate a higher presence of MHC type I fibers and in the male EDL compared to controls, areas of green were also noted, indicating MHC Type IIa fibers. These changes would be expected to contribute to reduced force generation based on the presence of more slow-twitch (oxidative) fiber types, which have slower speeds of contraction and thus overall lower force/power outputs. A similar trend is also observed in the SOL muscles, where in general there seems to be more MHC type I muscle fibers in the Sost-CreER^T2^ muscles with the SOL male muscles also displaying a few red MHC type IIb (fast twitch, glycolytic) muscle fibers. While the few IIb could increase force output, very likely the other structural changes, such as those indicating higher amounts of collagen, might also account for the overall force decrement.

**Fig. 7:**
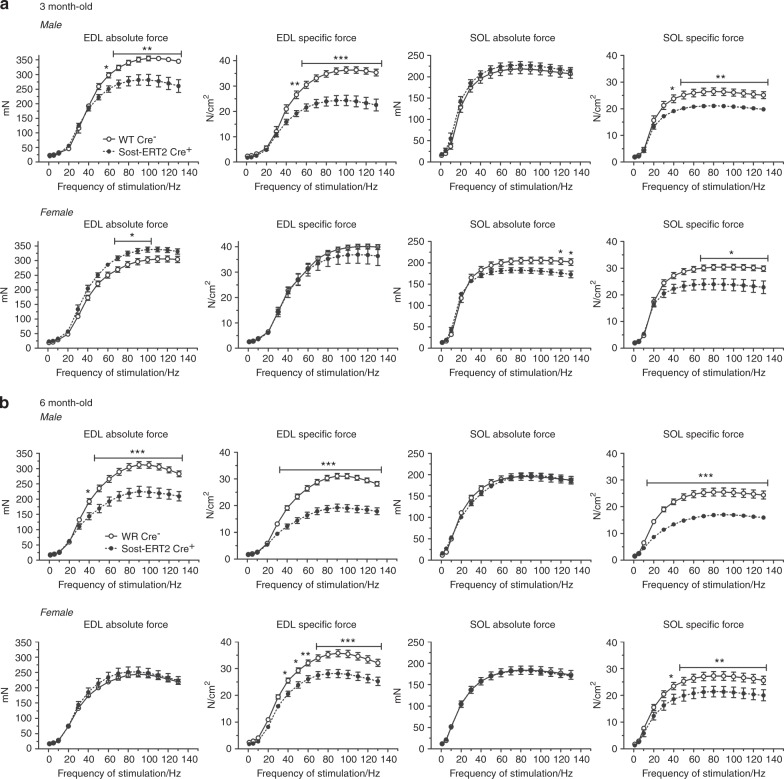
Muscle function of 3 and 6 mo male and female Cre^−^ and Cre^+^ animals. EDL and SOL muscle force vs. stimulation frequency analysis. **a** Absolute and specific force for EDL and SOL in 3-month-old male and female Cre^–^ and Cre^+^ (Sost-CreER^T2^) mice (*n* = 6). **b** Absolute and specific force for EDL and SOL for 6-month-old male and female Cre^–^ and Cre^+^ mice (*n* = 10). *P < 0.05, **P < 0.01, ***P < 0.001 vs. Cre^−^ using two way ANOVA with Bonferroni post-hoc analysis

Concerning muscle function, differences in absolute and specific force produced at different frequencies of stimulation of EDL and SOL muscles for 3 and 6 month old males and females are shown in Fig. 7. As would be expected based on the disruption in muscle histology, male Sost-CreER^T2^ EDL and SOL display reduced specific force at submaximal and maximal frequencies (from 20 to 130 Hz) at both 3 months (Fig. 7a) and 6 month (Fig. 7b) compared to Cre^−^ controls. Females displayed a reduction in specific force in Sost-CreER^T2^ SOL muscles at both 3 (Fig. 7a) and 6 months (Fig. 7b), and EDL specific force at 6 months, but not 3 months compared to Cre^−^ controls. In terms of absolute force, Sost-CreER^T2^ EDL muscles from males at 3 and 6 months displayed a decrease in absolute force compared to Cre^−^ controls, but there was no difference in SOL at either age. Sost-CreER^T2^ EDL muscles from females at 3 months showed increased force at submaximal frequencies, but at 6 months there was no difference compared to Cre^−^ controls. Female Sost-CreER^T2^ SOL muscles at 3 months showed a decrease in absolute force at maximal frequencies, but no change at 6 months compared to Cre^−^ controls.

To determine at what age this muscle phenotype was occurring, RNAseq was performed on gastrocnemius muscle since the EDL and SOL were already being utilized for contractile studies. The gastrocnemius is a mixed muscle type also located in the hindlimb and therefore was a good muscle to reflect both fast and slow twitch muscle fiber types. Since changes in the SOL and EDL muscle force parameters were apparent earlier in males than females, the time point selected for RNAseq were 1 mo old in males and 3 mo old in females. Already at one month of age, highly significant differences were observed in the muscle of male mice and few differences in the 3 mo female mice compared to controls. (See Fig. 1 supplemental data). In the 1 mo male mice there were 1 857 2-fold upregulated genes and 1 959 downregulated genes, whereas in 3 mo females there were 37 upregulated and 34 downregulated genes (Supp Fig. 1A). Only 14 upregulated and 21 downregulated genes overlapped between males and females. The overlapping genes are shown in Supplementary Table 1 and Table 2. A pathway analysis of the male gastrocnemius was performed showing that inflammation mediated by chemokine and cytokine signaling pathways was the major upregulated pathway in Cre^+^ as compared to Cre^–^ male mice (Supp. Figure 1B). The inflammatory related genes upregulated in Cre^+^ male mice are shown in Supplementary Table 3. The Wnt signaling pathway was the major downregulated pathway in the Cre^+^ male muscle. The GEO accession number for the RNAseq data is GSE119761.

**Fig. 8:**
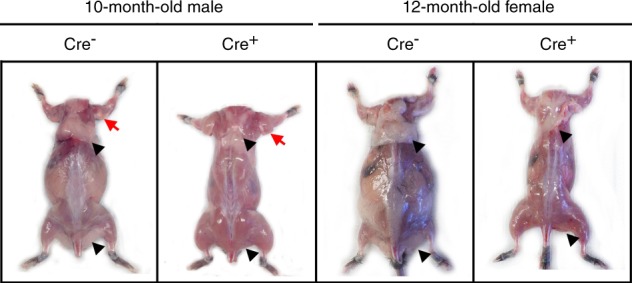
Effects of aging on muscle phenotype of Sost-CreER^T2^, Cre^+^, mice compared to Cre^–^ age matched controls. Representative images of a 10-month-old male and a 12-month-old female control Cre^–^ mouse and Sost-CreER^T2^, Cre^+^, mice without skin. Black arrow heads indicate fat tissue around the scapula and hind limb. Red arrow indicates larger triceps in 10-month-old male mice

Sost-CreER^T2^ Cre^+^ animals sacrificed at 10–12 mo of age had larger muscles and less fat than controls (Fig. 8). In 12 mo old females, a significant decrease in body size was observed in Cre^+^ mice compared to Cre^−^ controls (Fig. 8).

## Discussion

As there is a critical need for additional osteocyte selective Cre models, we generated a new Cre ER^T2^ model, driven by the Sost promoter. Our hypothesis was that Sost ER^T2^ Cre would be expressed only in osteocytes in bone and not in other tissues to any significant extent. The specificity of this mouse model was impressive with regards to targeting the mature osteocyte. No activation was ever observed in muscle, bone marrow, or bone surface cells. Spontaneous activation of Cre in the absence of tamoxifen only occurred in a few mature osteocytes in bone. With tamoxifen injection, this specificity was still mainly targeted to osteocytes, however, a small amount of off target activity was observed in a few cells in the retina, heart, brain, and kidney. Similar to other studies, we did not observe any sclerostin protein expression in these tissues compared to the high expression in osteocytes.^28^ Therefore, compared to other Cre models used for targeting osteocytes, this Cre model has a very high specificity for the mature osteocyte.

This goal of generating an osteocyte specific targeted Cre was accomplished but as frequently occurs with transgenic mouse models, an unexpected phenotype was observed. By X-ray and micro CT no significant differences were observed in bone parameters between Cre positive and Cre negative mice. However, with time, it appeared that the muscles in these animals were becoming larger as viewed by X-ray imaging. By 8–10 months, the muscle phenotype was obvious in Sost ER^T2^ Cre males and by 12 months in females. Even though a visual difference in the intact animals or their muscle appearance was not obvious at 6 months, when excised, the muscles were larger and abnormalities were evident based on histology and fiber typing. Functional studies showed that the muscles for the Cre positive mice were considerably weaker than controls.

Careful systematic analyses of the Sost ER^T2^ Cre mice revealed a pattern compatible with development of partial muscle myopathy. Healthy muscle has polygonal shaped fibers, relatively small variation in size, peripheral nuclei, no fiber splitting, and scant endomyosial collagen.^29^ Myopathic muscle is characterized by more central nuclei and they are scattered, atrophic, grouped, rounded fibers, with significantly more collagen around them indicating fiber replacement by fibrosis and can show fiber splitting.^29^ While many of these characteristics are clear in the Sost ER^T2^ Cre positive muscles, they are not atrophic but rather display possible compensatory hypertrophy, perhaps in adaptation to the recurring degeneration/regeneration leading to much larger muscle sizes. Muscle hypertrophy of this type is commonly seen in dystrophic muscle.^30^

At the functional level, the reduced muscle specific force supports the observed findings of remodeling and abnormal structure in the histological and immunohistochemical analysis. The larger muscles did not translate into improved muscle function, and in fact, normalized forces taking into account muscle size over a wide range of frequencies of stimulation were reduced in the Sost ER^T2^ Cre of both genders in both EDL and SOL muscles, demonstrating that the myopathy-like phenotype impaired the force generating capacity of the contractile machinery. This is yet another example that muscle size is not the most reliable parameter for muscle related studies, but rather muscle quality, and the need to always match muscle size to function.^31,32^ It is interesting that these muscles share some similarities between regenerating muscle fibers and dystrophic muscle fibers.^33^ Our RNA-Seq data is supportive of these ideas. Dystrophin expression was reduced (approximately 30 fold decrease in males and 2.5 fold decrease in females) and the observed phenotype seems to mimic the absence of dystrophin in Duchenne Muscular Dystrophy. The loss of dystrophin would invariably make skeletal muscles more fragile and more prone to damage. This damage may lead to inflammatory responses as well as to muscle trying to generate new muscle fibers. We observed significant expression of inflammatory genes which could be the underlying cause leading to the muscle remodeling and myopathy. A significant overexpression of myh8, a perinatal isoform of MHC as well as mhc3, an embryonic isoform, was also observed. These MHC isoforms normally re-express in regenerating muscles.

Intriguingly, *MUSK* (Muscle Associated Receptor Tyrosine Kinase), a gene that plays essential functions in the maintenance of the neuromuscular junction is equally down-regulated in both male and females. Mutations in this gene associate with congenital Myasthenia Gravis. It is also interesting to note that there were lower levels of myostatin and FKBP5 (leading to calcineurin activation) which can lead to muscle growth. One possibility would be that reduced MUSK and dystrophin expression lead to remodeling of the muscle and adaptations of growth to compensate, but these changes ultimately lead to myopathy. It remains to be determined the molecular mechanisms that induce these changes and how they interplay together to lead to the muscle phenotype.

It is also intriguing that in older Sost ER^T2^ Cre mice reaching their middle age (10–12 months), there seems to be a natural protection against fat accumulation, while muscle size still appears to be macroscopically larger, perhaps a reflection of lipid and insulin signaling regulatory changes. It might be interesting to determine if the myopathy persists during aging and if these larger (but disturbed, myopathic) muscles confer any protection against sarcopenia and osteopenia.

In summary, a novel inducible Cre model has been generated for targeting osteocytes, the Sost ER^T2^ Cre. This model shows high specificity for osteocytes. However, a low level of spontaneous Cre activation was seen that increased with age, but only in osteocytes. A muscle phenotype was also observed that increased with aging. Care should be exercised when using this model and the muscle phenotype taken into consideration for any future studies.

## Materials And Methods

### Generation of mice

In the Cre-ER^T^ system, the Cre recombinase sequence is fused with a mutated ligand-binding domain from the progesterone or estrogen receptor (ER).^8^ This modified ligand-binding domain becomes localized to the nucleus in the presence of synthetic steroids, such as tamoxifen or 4-hydroxy-tamoxifen. The Cre-ER^T^ system allows widespread time-dependent recombination when the Cre-ER^T^ activity is placed under the control of a strong promoter. The Cre-ER^T2^ is 10-fold more sensitive to 4-hydroxy-tamoxifen than the Cre-ER^T^ model.^34^

The Bac clone RP24-178J4, 157Kb was used for making the Sost Cre ER^T2^. Using standard recombineering techniques, 5’ and 3’ homology arms of about 300pb were prepared such that the 50 bp around the ATG initiation codon will be deleted. The arms were cloned into the CreERt2-frt-kanamycin-frt cassette that was put into the plasmid, PL451.^35^ The plasmid was then electroporated into SW105 competent cells, positive clones resistant to kanamycin-chloramphenicol were selected as described in Warming et al.^35^ and protocols supplied by the Neil Copeland lab. The kanamycin cassette was removed by treatment with L-arabinose that activates the flip recombinase. Positive clones were identified by PCR with appropriate primers, and BAC DNA purified with Marligen Plasmid Midprep kits. The BAC DNA was then sent to Transgenic Technology Center at UT Southwestern at Dallas, and injected into C57BL/6 N fertilized eggs. Forty-six tail DNA preparations were screened by Southern analysis and PCR and 13 lines were identified that were positive by both Southern and PCR. 4 of the 13 lines were crossed with the Rosa26-loxP-stop-loxP-tdTomato and frozen sections analyzed with red fluorescence.

The founder lines were crossed with Ai9 tdTomato reporter mice (B6;129S6-*Gt(ROSA)26Sor*^*tm9(CAG-tdTomato)Hze*^/J, reference 007905, The Jackson Laboratory, Bar Harbor, Maine, USA) in order to study the spatial and temporal recombination in osteocytes to identify the best line responding with high recombination after tamoxifen injection. The Ai9 mice harbor a targeted mutation of the *Gt(ROSA)26Sor* locus with a *loxP*-flanked STOP cassette preventing transcription of a CAG promoter-driven red fluorescent protein variant (tdTomato), and are useful as a Cre reporter strain. TdTomato is expressed following Cre-mediated recombination. Ai9 mice hemizygous for this Rosa-CAG-LSL-tdTomato-WPRE conditional allele are viable and fertile. When bred to mice that express Cre recombinase, the resulting offspring will have the STOP cassette deleted in the *cre*-expressing tissue(s); resulting in expression of tdTomato (Jackson Lab). These mice have been previously used to characterize numerous Cre models.^9^

All animal experiments were performed according to an approved Institutional Animal Care and Use Committee protocol at the University of Missouri Kansas City (UMKC), conforming to relevant federal guidelines. The UMKC animal facility is operated as a specific pathogen-free, AAALAC approved facility. Animal care and husbandry meet the requirements in the Guide for the Care and use of Laboratory Animals (8^th^ edition), National Research Council. Animals are group housed and maintained on a 12 h light/dark cycle with ad libitum food and water at a constant temperature of (23 ± 2) °C. Qualified veterinary staff and/or animal care technicians perform daily health check inspections.

### DNA extraction and genotyping

At ten days of age, (or two days of age when sacrificing pups), a piece of tail and toe was taken and digested in 500 µL 0.1 mol·L^−1^ Tris, 5 mmol·L^−1^ EDTA, 0.2% SDS and 0.2 mol·L^−1^ NaCl. Proteinase K was added at a final concentration of 0.2 mg·mL^−1^. The samples were incubated at + 50 °C overnight. The samples were then vortexed and microfuged at 13 K r·min^−1^ (Eppendorf 5415D, Hauppauge, NY, USA). DNA was precipitated from the supernatant using an equal volume of 2-propanol (HPLC grade, Fisher Scientific, Hanover Park, IL, USA). After centrifugation, the pellet was washed with 1 mL ethanol (ethanol 200 proof, Decon Laboratories Inc., King of Prussia, PA, USA) and re-centrifuged for removal of supernatent. The samples were dried and resuspended in a buffer containing 10 mmol·L^−1^ Tris and 1 mmol·L^−1^ EDTA. The amount and quality of DNA present in the buffer was quantitated using a NanoDrop device and software (NanoDrop 2000 spectrophotometer, Thermo Scientific, Waltham, MA USA). The DNA was stored at −20 °C.

To genotype, the samples were allowed to thaw at room temperature. TagMix (Redtaq® Ready Mix PCR, Sigma, St. Louis, MO, USA) was mixed with primers forward and reverse for Cre. The sequences are the following Cre-Frd 5’-CCG GGC TGC CAC GAC CAA GT-3’, Cre-Rvz 5’-GCG CGA GTT GAT AGC TGG CTG GT-3’, Sost-Fwd 5’-CAC TGC GGG CTC TAC TTC AT-3’, Sost-Rvz 5’-TCT TCA TCC CGT ACC TTT GG-3’, td tomato WT sequence: 01MR9020: 5’-AAG GGA GCT GCA GTG GAG TA-3’, 01MR9021: 5’-CCG AAA ATC TGT GGG AAG TC-3’. The td tomato mutant sequences are 01MR9103; 5’-GGC ATT AAA GCA GCG TAT CC-3’ and 01MR9105: 5’-CTG TTC CTG TAC GGC ATG G-3’. The primers were ordered from IDT (Coralville, IO, USA). One microliter of DNA was added to 24 μL of the mix. PCR was done using PTC-200 (MJ Research, St. Bruno, Quebec, Canada) and i-cycler (Biorad Laboratories Inc., Hercules, CA, USA) machines to amplify the DNA sequence of interest. 1.2% agarose gels (Biorad Laboratories Inc., Hercules, CA, USA) were used to separate DNA. The genotyping (PCR and gel) was repeated two times minimum to confirm the genotype for each mouse.

### Tamoxifen injections

Tamoxifen was purchased from Sigma (T5648, Sigma-Aldrich, St. Louis, MO, USA) and diluted in sterile Corn Oil (C8267, Sigma-Aldrich, St Louis, MO, USA). The solution was sonicated for 15 min to allow the powder to mix with the oil. 2 mo and 5 mo old male and female Sost ER^T2^ Cre^+^ x td tomato and Sost ERT2 Cre^–^ x td tomato mice were injected i.p. with tamoxifen up to 250–550 mg·kg^−1^ total dose using a concentration of 20 mg·mL^−1^ as advised by Jackson Laboratories. The mice received 5 to 6 injections on consecutive days to achieve this total dose. The dose injected was never more than 100–150 μL per day. Sost ER^T2^ Cre^+^ and Sost ER^T2^ Cre^–^ control mice were injected with the same volume of corn oil vehicle.

### Epifluorescence microscopy

Before tamoxifen injections, at 2 and 5 months of age, a tail vertebra was removed under anesthesia (isofluorane) in order to quantitate the basal amount of Cre recombination in bone osteocytes. Four weeks later after tamoxifen injections, at 3 and 6 months of age, the animals were sacrificed and bones and soft tissues were analyzed as described below.

At sacrifice, bones (right femur, and tail vertebrae), soft tissues and glands (brain, kidney, heart, liver, lung, thymus, muscle, stomach, skin, small intestine, large intestine, spleen, eye, gallbladder, urinary bladder, ovaries, testis, uterus, vagina, pancreas, tongue, trachea, oesophagus, aorta, mammary glands, pituitary gland, lacrimal gland, salivary glands) were dissected free of fat, skin, muscle and connective tissue and fixed in 4% PFA for 1 day at + 4 °C. The bones were placed in 14% EDTA at room temperature in the dark for 1 week for decalcification. Organs were washed with 1x PBS three times for 15 min and incubated in 15% sucrose for 4 h, and sucrose 30% overnight. The next day, organs were embedded in OCT at −20 °C (optimal cutting temperature, TissueTek, Sakura Finetek, Torrance, CA, USA). After 1 week in EDTA, bones were washed and treated with 15% and 30% sucrose, then embedded as described above. Ten micron thick cryosections of bones and soft tissues were cut using a cryostat (Leica CM3050S, Buffalo Grove, IL, USA), with cryotape (Cryofilm type IIc(10), Section Lab Co Ltd., Hiroshima, Japan), and placed on positively charged microscope slides (Superfrost Plus, Shandon, Thermo Scientific, Waltham, MA, USA). Slides were mounted with DAPI mounting media (Fluoroshield^™^ with DAPI, Sigma, St Louis, MO, USA) and imaged with an epifluorescence microscope (Eclipse E800, Nikon, Tokyo, Japan), configured with an Optronics CCD color camera (Optronics, Goleta, CA) and interfaced with the AnalySIS software (Soft Imaging System GmbH, Muenster, Germany). For fluorescence imaging a TRITC filter (excitation 530–550, emission 590–650) and a DAPI filter (excitation 330–380, emission 435–485) were used. Exposures were 125 ms for TdTomato, 125 and 250 ms for DAPI at 4x and 20x and 2 and 50 ms for brightfield at 4x and 20x magnifications respectively.

### Quantitation of positive osteocytes

Brightfield, DAPI and TdTomato images were taken with 4x and 20x magnifications. The 20x magnification was used to quantitate osteocyte number. The number of osteocytes stained with DAPI and the number that were positive for tdTomato fluorescence were quantitated on 4 images per animal using Image J software (v1.48 v: Wayne Rasband, National Institutes of Health). The percentage of positive osteocytes was calculated for each field and then averaged for each mouse.

### Determination of spontaneous Cre activity

To determine spontaneous (non-tamoxifen induced) Cre activity also termed ‘leakiness’, non-tamoxifen injected animals were sacrificed at different ages. At day 2, pups were sacrificed by CO_2_ inhalation and genotyped as described above. The pups were cut longitudinally, placed in cryomolds with OCT and submersed in isopentane (AC12647–0010, Fisher scientific, Hanover Park, IL, USA) in liquid nitrogen, in order to preserve the tissue. The frozen samples were then stored at −80 °C. Ten micron thick sagittal cryosections of the pups were prepared using cryotape. Animals at 1, 2, and 5 months were also examined for ‘leakiness’ using the 3^rd^ to 5^th^ caudal tail vertebrae.

### Confocal microscopy

For confocal imaging, sections were imaged on a Leica TCS Sp5 II confocal microscope (Leica Microsystems, Wetzlar, Germany) in resonant scanner mode using LAS-AF software version 3.0.0 for TCS SP8. For imaging tdTomato, laser excitation was 543 nm with a collection window of 553–650 nm and for imaging DAPI, laser excitation was 405 nm with a collection window of 410–480 nm. Imaging was done with a 20x objective (NA 0.7, zoom 1.7, single Z plane) and a 100x oil objective (NA 1.44 zoom 1.7, 25 Z planes with a 0.17 µm step size).

### Immunostaining for sclerostin

Cryosections (10 µm thick) from eye, lung, heart, and brain were cut as described above. Endogenous peroxidase was quenched with 3% hydrogen peroxide in PBS for 10 min in the dark, then washed with PBS 3 times. Blocking was done with 3% BSA and 10% rabbit serum in PBS for 1 h at room temperature. Then primary goat anti-mouse sclerostin polyclonal antibody (AF 1589, R&D, Minneapolis, MN, USA) was diluted at concentrations of 1:100 and 1:300 in PBS + 10% rabbit serum and added on the slides. Goat IgG was used as a negative control. Bone was used as a positive control (1:50, 1:100, 1:200, 1:500, 1:1 000 tested). The primary antibody was incubated at + 4 °C overnight. The next day, the primary antibody was washed 3 times with PBS and secondary antibody (rabbit anti-goat) was prepared in PBS + 2% rabbit serum and added to the sections for 1 h at room temperature. The secondary antibody was washed with PBS 3 times and detection was done using the Vector ABC peroxidase kit (Vector PK-4000, Vector Laboratories, Burlingame, CA), according to manufacturer’s instructions. The sections were then incubated 3 min with a substrate containing 0.5 mg·mL^−1^ DAB (3,3’ diaminobenzidine tetrahydrochloride, Sigma, D5637) in 50 mmol·L^−1^ Tris.Cl, distilled water and 30% hydrogen peroxide (final concentration 0.05%), pH 7.4. Sections were counterstained with methyl green and the slides were dehydrated and mounted in permount. Images were collected on a Nikon Eclipse E800 microscope (Nikon, Tokyo, Japan) with brightfield illumination using an Optronics CCD color camera (Optronics, Goleta, CA) interfaced with the AnalySIS software (Soft Imaging System GmbH, Muenster, Germany).

### Immunostaining for muscle fiber typing

Immunostaining was performed as described by Fry et al.^36^ Extensor digitorum longus (EDL; primarily glycolytic, fast twitch muscle) and Soleus (SOL; primarily oxidative, slow twitch muscle) were dissected then embedded in OCT compound and rapidly frozen in isopentane in liquid nitrogen. Transverse cryosections, 14 microns thick, were cut and placed onto glass slides. The sections were fixed by acetone for 5 min and washed 3 times for 3 min. Blocking was performed using M.O.M. mouse IgG blocking reagent (MKB-2231, Vector Laboratories) for 1 h, and the sections were then washed 3 times for 3 min. The primary antibody for mouse dystrophin (1:150) (VP-D505, Vector Laboratories) diluted in 2% goat serum/PBS was applied to sections and incubated overnight at 4 °C in a humidified chamber. The sections were again washed 3 times for 3 min in PBS followed by incubation with Goat anti-mouse biotin secondary antibody (Biotin-SP-AffiniPure goat anti-Mouse IgG 115065–205, Jackson Immunoresearch, West Grove, PA, USA) for 1.5 h at RT, again washing with PBS before application of fluorophore (AMCA-Streptavidin, Vector Laboratories) diluted with PBS (1:150) 1 h at RT. This was followed by washing with PBS, blocking again with M.O.M. for 30 min at RT and washing with PBS. Then the sections were incubated overnight at 4 °C in mouse monoclonal antibodies for MHCs diluted in 2% goat serum/PBS. These were obtained from the Developmental Studies Hybridoma Bank at the University of Iowa, IA, USA and included antibodies to Type I fibers (BA.D5 mouse IgG2b, 1:100); Type IIa (SC.71 mouse IgG1, 1:200); and Type IIb (BF.F3 mouse IgM, 1:100). The sections were then incubated with secondary antibodies diluted at 1:250 in 2% goat serum/PBS for 1 h at RT. These included an Alexa Fluor 647 conjugated goat anti-mouse IgG2b; an Alexa Fluor 488 conjugated goat anti-mouse IgG1; and an Alexa Fluor 594 conjugated goat anti-mouse IgM (Life Technologies, Carlsbad, CA). After washing with PBS, the sections were post-fixed with methanol for 5 minutes at RT, washed and coverslip mounted with mounting media (H-1000, Vector Laboratories). The samples were imaged as described above with a Nikon Eclipse E800 epifluorescence microscope using excitation and emission filters optimized for each fluorophore.

### X-ray imaging

On the day of sacrifice, the mice were anesthetized with ketamine/dex-dormitor (75 mg·kg^−1^, 0.5 mg·kg^−1^), injected i.p. (100 μL per 10 g body weight). The mice were then X-rayed using a Faxitron MX-20 × -ray cabinet (Faxitron, Tucson, AZ, USA) at 2x magnification and 28 kVP for 10 s in order to obtain X-rays of the whole body on the ventral and lateral sides.

### Microcomputed tomography

Micro CT analysis was performed on the tibia in control wild type and Sost ER^T2^ Cre mice at 6-months of age (*n* = 4 for male and *n* = 5 for female). Micro CT scanning was conducted using a Scanco viva CT40 (Scanco Inc, Basel, Switzerland) at a resolution of 10 µm, 55 kV voltage, 145 µA current, and 200 ms integration time following recommended guidelines.^37^ Analysis was performed on tibiae fixed in 70% ethanol. Using our standard approach for adult bones, a high-resolution scan of the region of interest was performed. The analysis was performed 2.0 mm to 3.0 mm from the growth plate for trabecular bone and 1.0 mm to 1.5 mm above the junction of tibia and fibula for cortical bone. The threshold for trabecular and cortical bone was set to 270 and 355, respectively. Three-dimensional analyses were performed to determine the trabecular bone volume/tissue volume (Tb.BV/TV), trabecular number (Tb.N), trabecular thickness (Tb.Th), trabecular separation (Tb.Sp) at the proximal tibia. For cortical bone analysis, bone volume/tissue volume (Ct.BV/TV), and cortical bone thickness (Ct.Th) were determined.

### Ex vivo skeletal muscle function

These experiments were performed as previously described.^31,38^ Intact EDL and SOL muscles were dissected from 3 and 6 month old mice. Muscles were ligated to a force transducer and a fixed support by the tendons and placed inside Radnotti Chambers containing oxygenated Ringer’s solution (in mmol·L^−1^; 142 NaCl, 5 KCl, 1.8 MgCl_2_, 2.5 CaCl_2_, 10 HEPES, 10 glucose; pH 7.4) for the collection of isometric force data using an 8-chamber system. Experiments were driven by an ADI-PowerLab Software (Colorado Springs, CO, USA) that is customized for these experiments. Stimulation voltage (100 V; 500 ms trains; 1 ms pulse) was supplied by a Grass S88X stimulator and contractile measurements were obtained at room temperature. Muscles were first subjected to the length-force relationship analysis to determine the optimal length (Lo) at which maximal force is achieved. Next, muscles were equilibrated to mimic conditions of normal activity (low duty cycle). Following 20 min equilibration, muscles were stimulated with frequencies ranging from 1–130 Hz to analyze the force *versus* frequency relationship. After contractile measurements, muscle Lo was measured and muscles were excised at the myotendonous junctions, blotted dry and weighed for determination of specific force to account for muscle size through the following formula:

$$\frac{{Force\left( N \right) \cdot Lo\left( {cm} \right) \cdot 1.06\left( {\frac{g}{{cm^3}}} \right)}}{{Muscle\;Mass(g)}} = N/cm^2$$

### RNAseq analysis

Gastrocnemius muscle was isolated from 1 mo old male and 3 mo old female control wild type and Sost ER^T2^ Cre mice. Total RNA was extracted from grastrocnemius muscles using a Fibrous Tissue RNeasy Midi Kit (Qiagen). Further RNA cleaning was performed with an RNeasy MinElute Cleanup Kit (Qiagen). Gastrocnemius muscles from both legs were combined for RNA extraction and RNA was stored at −80 °C until RNAseq analysis.

*Library Preparation and sequencing*. The concentration and quality of total RNA samples were first assessed using Agilent 2100 Bioanalyzer. A RIN (RNA Integrity Number) of five or higher was required to pass the quality control. Then five hundred nanograms of RNA per sample was used prepare dual-indexed strand-specific cDNA library using TruSeq Stranded mRNA Library Prep Kit (Illumina). The resulting libraries were assessed for its quantity and size distribution using Qubit and Agilent 2100 Bioanalyzer. Two hundred picomolar pooled libraries were utilized per flowcell for clustering amplification on cBot using HiSeq 3000/4000 PE Cluster Kit and sequenced with 2 × 75 bp paired-end configuration on HiSeq4000 (Illumina) using HiSeq 3000/4000 PE SBS Kit. A Phred quality score (Q score) was used to measure the quality of sequencing. More than 90% of the sequencing reads reached Q30 (99.9% base call accuracy).

*Sequence alignment and gene count*. The sequencing data were first assessed using FastQC (Babraham Bioinformatics, Cambridge, UK) for quality control. Then all sequenced libraries were mapped to the mm10 mouse genome using STAR RNA-seq aligner^39^ with the following parameter: “--outSAMmapqUnique 60”. The reads distribution across the genome was assessed using bamutils (from ngsutils).^40^ Uniquely mapped sequencing reads were assigned to mm10 RefSeq genes using featureCounts (from subread)^41^ with the following parameters: “-s 2 -p –Q 10”. Quality control of sequencing and mapping results was summarized using MultiQC.^42^ Genes with read count per million (CPM) > 0.5 in more than 2 of the samples were kept. The data were normalized using TMM (trimmed mean of M values) method. Differential expression analysis was performed using edgeR.^43,44^ False discovery rate (FDR) was computed from p-values using the Benjamini-Hochberg procedure.

*Pathway Analysis*. Venn diagrams (http://bioinformatics.psb.ugent.be/webtools/Venn/) were created using 2 fold up- and down-regulated genes with FDR < 0.05 compared to wild-type. The identified shared genes in both genders are listed in Supplemental Tables 1 and 2. 5 fold up- and down-regulated genes compared to wild-type were analyzed by Protein ANalysis THrough Evolutionary Relationships (PANTHER) which is a freely available, comprehensive classification system (http://www.pantherdb.org/).^45^ The PANTHER Overrepresentation Test (Released 2017–12–05) was used to search the data against the PANTHER database (PANTHER version 13.1 Released 2018–02–03) to identify pathways overrepresented in our data when compared to a reference mouse genome. The analysis is performed by a Fisher’s Exact Test in combination with a robust False Discovery Rate (FDR) correction for multiple testing. The pathway classification and gene list are provided in the Supplemental Fig. 1b and Table 3.

## Electronic supplementary material

Supplementary Figure 1

Supplementary Table 1

Supplementary Table 2

Supplementary Table 3

Supplementary Information
