# Supplementary material for: Characterization of a novel murine Sost ERT2 Cre model targeting osteocytes
Source: Bone Res. 2019 Feb 21;7:6. doi: 10.1038/s41413-018-0037-4 (PMC6382861; doi:10.1038/s41413-018-0037-4)

Supplementary Figure 1

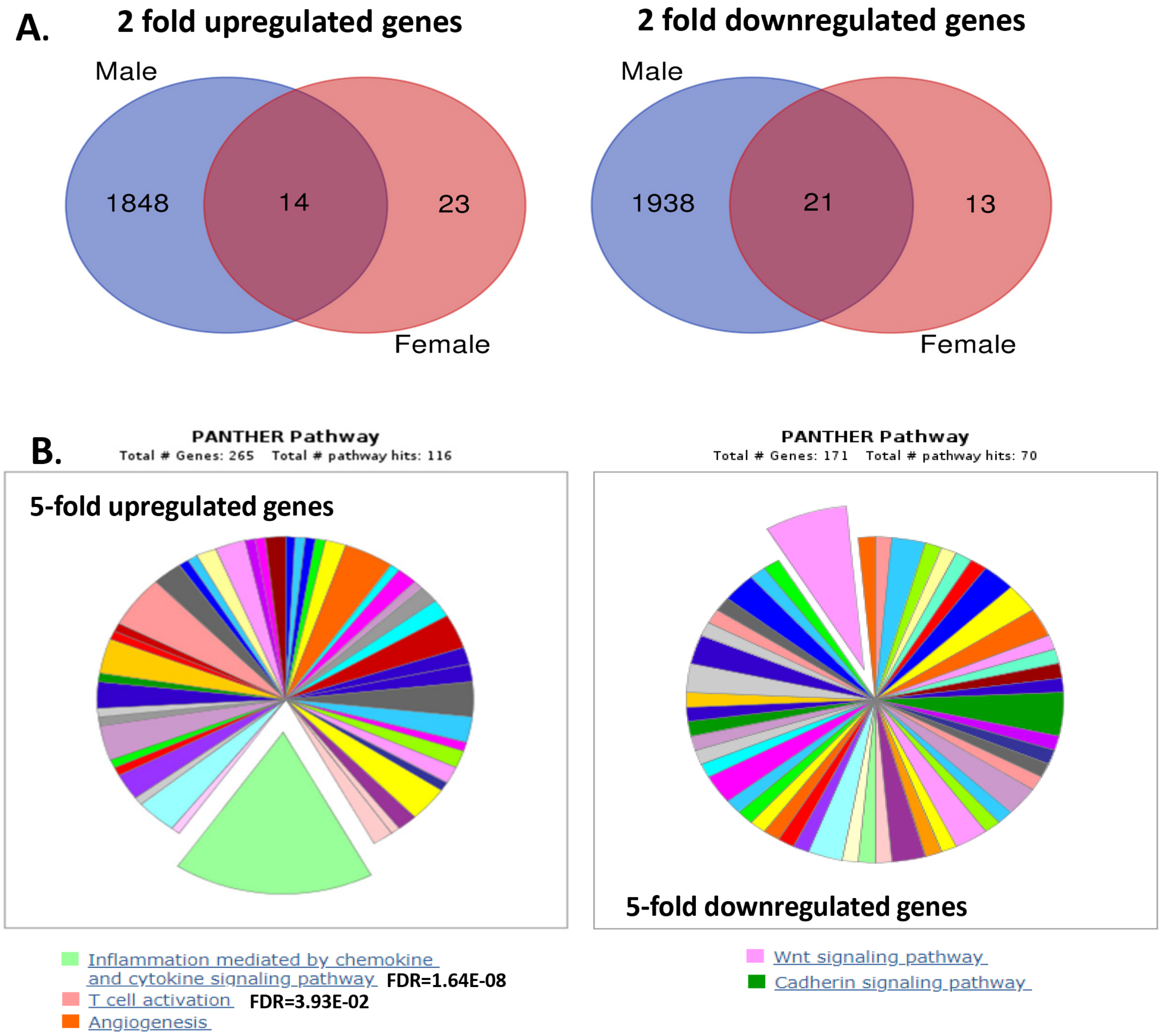

Supplement: Supplementary file 1 — Supplementary Figure 1 [file 41413_2018_37_MOESM1_ESM.pdf]
