# Supplementary material for: Characterization of a novel murine Sost ERT2 Cre model targeting osteocytes
Source: Bone Res. 2019 Feb 21;7:6. doi: 10.1038/s41413-018-0037-4 (PMC6382861; doi:10.1038/s41413-018-0037-4)
Supplement: Supplementary file 2 — Supplementary Table 1 [file 41413_2018_37_MOESM2_ESM.pdf]

Supplementary Table 1: 2 fold upregulated genes in both male and female gastrocnemius skeletal muscles.

Gene Symbol	Gene Name	Male: SOSTER2Cre vs WT				Female: SOSTER2Cre vs WT			
		Fold Change	log2FC	PValue	FDR	Fold Change	log2FC	PValue	FDR
5430431A17Rik		**5.3301237**	2.414169	7.71E-18	5.28E-16	**2.161197**	1.111831	0.000202	0.029913
Golga7b	Golgin subfamily A member 7B	**5.1141334**	2.35449	3.71E-11	4.37E-10	**2.6628236**	1.412957	0.000153	0.025753
Wfdc17	Activated macrophage/microglia WAP domain protein	**4.729846**	2.241793	7.62E-25	2.1E-22	**2.0487446**	1.03474	0.000507	0.049864
Spint2	Kunitz-type protease inhibitor 2	**4.217001**	2.076217	5.42E-16	2.31E-14	**2.269239**	1.182209	2.64E-05	0.009548
Vps37d	Vacuolar protein sorting-associated protein 37D	**3.8614774**	1.949153	2.76E-06	1.07E-05	**3.8742892**	1.953932	0.000417	0.044908
Exoc3l4	Exocyst complex component 3-like protein 4	**3.6369363**	1.862724	8.58E-22	1.49E-19	**2.6004857**	1.378781	1.27E-07	0.000256
Cpxm1	Probable carboxypeptidase X1	**2.9831074**	1.576816	2.19E-12	3.48E-11	**2.0743611**	1.052667	2.94E-05	0.009866
Hp	Haptoglobin	**2.9665773**	1.568799	1.6E-11	2.03E-10	**2.0846849**	1.059829	3.28E-05	0.010496
Sncg	Gamma-synuclein	**2.6982326**	1.432015	1.06E-08	6.75E-08	**2.0338331**	1.024201	0.000126	0.022248
Col27a1	Collagen alpha-1(XXVII) chain	**2.618286**	1.388623	3.56E-09	2.54E-08	**2.1283233**	1.089717	0.000112	0.020832
Cdsn	Corneodesmosin	**2.5630368**	1.357854	0.028307	0.046299	**3.9035725**	1.964795	0.000266	0.03526
2700046G09Rik		**2.5076345**	1.326327	1.47E-08	9.18E-08	**2.0507537**	1.036154	0.000176	0.027207
Gkn3	Gastrokine-3	**2.3127879**	1.209633	0.001336	0.002958	**3.7394409**	1.902823	3.24E-06	0.00248
Gale	UDP-glucose 4-epimerase	**2.1806547**	1.124761	0.000344	0.000862	**2.4363926**	1.284747	0.000402	0.044908
