# Supplementary material for: Characterization of a novel murine Sost ERT2 Cre model targeting osteocytes
Source: Bone Res. 2019 Feb 21;7:6. doi: 10.1038/s41413-018-0037-4 (PMC6382861; doi:10.1038/s41413-018-0037-4)
Supplement: Supplementary file 3 — Supplementary Table 2 [file 41413_2018_37_MOESM3_ESM.pdf]

Supplementary Table 2: 2 fold downregulated genes in both male and female gastrocnemius skeletal muscles.

Gene Symbol	Gene Name	Male: SOSTER2Cre vs WT				Female: SOSTER2Cre vs WT			
		Fold Change	log2FC	PValue	FDR	Fold Change	log2FC	PValue	FDR
**Dmd**	**Dystrophin**	**-29.824117**	-4.89841	3.3E-57	2.27E-53	**-2.6070274**	-1.38241	3.79E-06	0.002606
Gbp10	Guanylate-binding protein 10	**-12.718009**	-3.6688	1.86E-08	1.14E-07	**-5.4607004**	-2.44909	0.000358	0.041749
**Mstn (GDF8)**	**Myostatin, Growth/differentiation factor 8**	**-11.057914**	-3.46701	2.22E-24	5.55E-22	**-2.3511303**	-1.23335	0.000475	0.048011
Gbp6	Guanylate-binding protein 6	**-6.1230768**	-2.61426	1.22E-13	2.77E-12	**-2.4954495**	-1.3193	8.01E-05	0.016216
Fkbp5	Peptidyl-prolyl cis-trans isomerase FKBP5	**-5.5428065**	-2.47062	8.17E-11	8.79E-10	**-3.3412337**	-1.74038	5.12E-05	0.012481
Eya4	Eyes absent homolog 4	**-5.0925665**	-2.34839	4.93E-22	9.29E-20	**-2.087705**	-1.06192	6.18E-05	0.014178
Fzd4	Frizzled-4	**-4.9701481**	-2.31329	2.29E-16	1.06E-14	**-2.4276045**	-1.27953	4.09E-05	0.011103
Txlng	Gamma-taxilin	**-4.2328581**	-2.08163	1.64E-14	4.84E-13	**-2.0977106**	-1.06882	0.000153	0.025753
Cmtr1	Cap-specific mRNA (nucleoside-2'-O-)-methyltransferase 1	**-4.1480776**	-2.05244	1.11E-08	7.1E-08	**-2.5998467**	-1.37843	6.45E-05	0.014262
Atp13a3	Probable cation-transporting ATPase 13A3	**-3.9475099**	-1.98094	1.04E-12	1.86E-11	**-2.1451963**	-1.10111	0.000414	0.044908
Slc40a1	Solute carrier family 40 member 1	**-3.7715247**	-1.91515	8.6E-14	2.04E-12	**-2.0116614**	-1.00839	0.000239	0.032517
Cd36	Platelet glycoprotein 4	**-3.685569**	-1.88189	3.58E-14	9.53E-13	**-2.1161795**	-1.08146	0.000101	0.019221
Mettl21e	Protein-lysine methyltransferase METTL21E	**-3.2002335**	-1.67818	7.93E-08	4.22E-07	**-2.6975421**	-1.43165	5.17E-05	0.012481
Nox4	NADPH oxidase 4	**-3.184863**	-1.67123	9.45E-08	4.96E-07	**-3.7366635**	-1.90175	8.87E-07	0.001087
Nbea	Neurobeachin	**-2.8270665**	-1.49931	6.67E-11	7.35E-10	**-2.1647411**	-1.11419	4.2E-05	0.011103
Cat	Catalase	**-2.7521112**	-1.46054	2.44E-12	3.84E-11	**-2.1291806**	-1.0903	3.52E-06	0.002549
Cdc14a	Dual specificity protein phosphatase CDC14A	**-2.6794811**	-1.42195	9.94E-13	1.78E-11	**-2.3181978**	-1.213	1.01E-07	0.000256
3110057O12Rik		**-2.6459436**	-1.40378	1.46E-06	5.98E-06	**-2.7224126**	-1.44489	1.37E-05	0.006077
Tmem100	Transmembrane protein 100	**-2.6254687**	-1.39257	2.02E-06	8.07E-06	**-2.195274**	-1.1344	0.000511	0.049864
Prrg1	Proline-rich Gla (G-carboxyglutamic acid) 1	**-2.1795649**	-1.12404	0.000199	0.000523	**-2.6000566**	-1.37854	0.000107	0.020075
**Musk**	**Muscle, skeletal receptor tyrosine-protein kinase**	**-2.1793576**	-1.1239	3.08E-06	1.19E-05	**-2.0473836**	-1.03378	0.000288	0.03707
