# Supplementary material for: Characterization of a novel murine Sost ERT2 Cre model targeting osteocytes
Source: Bone Res. 2019 Feb 21;7:6. doi: 10.1038/s41413-018-0037-4 (PMC6382861; doi:10.1038/s41413-018-0037-4)
Supplement: Supplementary file 4 — Supplementary Table 3 [file 41413_2018_37_MOESM4_ESM.pdf]

Supplementary Table 3: Inflammation-related genes upregulated in male gastrocnemius skeletal muscle.

Gene Symbol	Gene Name	Male: SOSTER2Cre vs WT				Female: SOSTER2Cre vs WT			
		Fold Change	log2FC	PValue	FDR	Fold Change	log2FC	PValue	FDR
Ccl4	C-C motif chemokine 4	**174.19128**	7.444529	8.04E-10	6.81E-09	**2.2033255**	1.139683	3.75E-01	0.662883
Ccl3	C-C motif chemokine 3	**160.8137**	7.329246	6.99E-11	7.66E-10	**1.4466868**	0.532753	0.737941	0.889174
Pf4	Platelet factor 4	**5.2050607**	2.379915	2.9E-16	1.31E-14	**-1.0411993**	-0.05825	0.854236	0.942731
Myh8	Myosin-8	**30.052684**	4.909422	1.23E-92	1.69E-88	**1.5624392**	0.6438	0.009273	0.146331
Ccl2	C-C motif chemokine 2	**27.164287**	4.763639	4.62E-10	4.16E-09	**1.5665813**	0.64762	0.436081	0.712258
Ccl22	C-C motif chemokine 22	**27.164287**	4.763639	4.62E-10	4.16E-09	**1.5665813**	0.64762	0.436081	0.712258
Ccl8	C-C motif chemokine 8	**21.976452**	4.457887	1.78E-32	1.36E-29	**1.178543**	0.237004	0.598476	0.812775
Ccl7	C-C motif chemokine 7	**16.376776**	4.033579	8.96E-13	1.62E-11	**2.8784375**	1.525286	2.17E-02	0.199236
Plcb2	1-phosphatidylinositol 4,5-bisphosphate phosphodiesterase beta-2	**9.9169393**	3.309895	8.65E-09	5.64E-08	**1.2035022**	0.267239	0.692426	0.867187
Ccl5	C-C motif chemokine 5	**8.6985258**	3.120771	2.26E-08	1.34E-07	**1.3328662**	0.414532	0.677954	0.858467
Myh3	Myosin-3	**8.7146133**	3.123437	1.07E-32	9.19E-30	**-1.2944247**	-0.37231	0.193602	0.491597
Rac2	Ras-related C3 botulinum toxin substrate 2	**8.3208204**	3.056726	6.94E-21	9.94E-19	**1.4827811**	0.568306	0.110883	0.382299
Vav1	Proto-oncogene vav	**7.0838837**	2.824541	1.23E-15	4.74E-14	**1.1921464**	0.253561	0.55916	0.789943
Ccr2	C-C chemokine receptor type 2	**5.3174547**	2.410736	1.07E-18	9.18E-17	**1.2206407**	0.287639	0.349458	0.641482
Itgb2	Integrin beta-2	**5.2947291**	2.404557	3.45E-17	2.03E-15	**-1.0024571**	-0.00354	0.991125	0.99747
Junb	Transcription factor jun-B	**5.6495228**	2.498129	8.1E-22	1.44E-19	**1.1373908**	0.185728	0.505641	0.762057
Ccl12	C-C motif chemokine 12	**5.532536**	2.467941	2.58E-05	8.15E-05	**-1.4765529**	-0.56223	0.461588	0.730471
Cxcl10	C-X-C motif chemokine 10	**5.2177127**	2.383418	6.58E-08	3.56E-07	**1.3832326**	0.468044	0.434558	0.711509
Alox5ap	Arachidonate 5-lipoxygenase-activating protein	**5.1059278**	2.352173	9.13E-20	9.81E-18	**1.0665107**	0.092898	0.743214	0.890822
Ptk2b	Protein-tyrosine kinase 2-beta	**5.0660339**	2.340857	3.82E-10	3.53E-09	1.388703	0.473738	0.274486	0.575331
