# Supplementary material for: Characterization of a novel murine Sost ERT2 Cre model targeting osteocytes
Source: Bone Res. 2019 Feb 21;7:6. doi: 10.1038/s41413-018-0037-4 (PMC6382861; doi:10.1038/s41413-018-0037-4)
Supplement: Supplementary file 5 — Supplementary Information [file 41413_2018_37_MOESM5_ESM.docx]

A title and description for each file.

1. Supplementary Figure 1

RNA-Seq analyses of 1 mo male and 3 mo female gastrocnemius skeletal muscles.

1. Supplementary Table 1:

2 fold upregulated genes in both male and female gastrocnemius skeletal muscles.

1. Supplementary Table 2:

2 fold downregulated genes in both male and female gastrocnemius skeletal muscles.

1. Supplementary Table 3:

Inflammation-related genes upregulated in male gastrocnemius skeletal muscle.
